# Plasticity of Mouse Dorsal Root Ganglion Neurons by Innate Immune Activation Is Influenced by Electrophysiological Activity

**DOI:** 10.1111/jnc.16292

**Published:** 2024-12-26

**Authors:** Timothy N. Friedman, Shawn M. Lamothe, Aislinn D. Maguire, Thomas Hammond, Gustavo Tenorio, Brett J. Hilton, Jason R. Plemel, Harley T. Kurata, Bradley J. Kerr

**Affiliations:** ^1^ Neuroscience and Mental Health Institute University of Alberta Edmonton Alberta Canada; ^2^ Department of Pharmacology University of Alberta Edmonton Alberta Canada; ^3^ Department of Anesthesiology and Pain Medicine University of Alberta Edmonton Alberta Canada; ^4^ Department of Cellular and Physiological Sciences, International Collaboration on Repair Discoveries (ICORD), and Djavad Mowafaghian Centre for Brain Health University of British Columbia Vancouver British Columbia Canada; ^5^ Division of Neurology, Department of Medicine University of Alberta Edmonton Alberta Canada

**Keywords:** DRG, electrophysiology, inflammation, Kv7 channels, neurite extension, pain, plasticity, TNFα

## Abstract

The complex relationship between inflammation, its effects on neuronal excitability and the ensuing plasticity of dorsal root ganglion (DRG) sensory neurons remains to be fully explored. In this study, we have employed a system of experiments assessing the impact of inflammatory conditioned media derived from activated immune cells on the excitability and activity of DRG neurons and how this relates to subsequent growth responses of these cells. We show here that an early phase of increased neuronal activity in response to inflammatory conditioned media is critical for the engagement of plastic processes and that neuronal excitability profiles are linked through time to the structural phenotype of individual neurons. Pharmacological blockade of neuronal activity was able to abolish the growth‐promoting effects of inflammatory media. Our results suggest that targeting the activity of DRG neurons may provide a novel therapeutic avenue to manipulate their growth status and potential for plasticity in response to inflammation. Importantly, the same pharmacological blockade in vivo abolished pain responses in a mouse model of multiple sclerosis. While further studies are needed to fully elucidate the underlying mechanisms of the relationship between neural activity and growth status, a more complete understanding of this relationship may ultimately lead to the development of new treatments for neuropathic pain in disorders associated with heightened immune responses such as rheumatoid arthritis and multiple sclerosis.

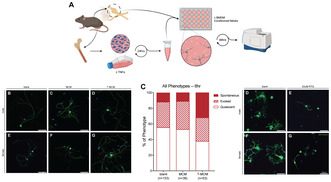

## Graphical abstract

We have carried out a series of experiments assessing the impact of inflammatory conditioned media derived from activated immune cells on the excitability and activity of dorsal root ganglia (DRG) neurons and how this relates to subsequent growth responses of these cells. We show here that an early phase of increased neuronal activity in response to inflammatory conditioned media is critical for later neurite outgrowth. DRG, Dorsal root ganglia.

**Abbreviations**
BSA: bovine serum albumin
CFA: complete Freund's adjuvant
CM: conditioned media
DMEM: Dulbecco's Modified Eagle's Medium
DMSO: dimethylsulfoxide
DPBS(Tx): Dulbecco's Phosphate Buffered Saline (Triton‐X)
DRG: dorsal root ganglia
EAE: experimental autoimmune encephalomyelitis
HBSS: Hank's Balanced Salt Solution
IL‐1B: interleukin‐1 beta
MCM: macrophage conditioned media
MOG: myelin oligodendrocyte glycoprotein
MS: multiple sclerosis
PFA: paraformaldehyde
RMP: resting membrane potential
RRID: Research Resource Identifier (see scicrunch.org)
RTG: retigabine
T‐MCM: TNF‐a macrophage conditioned media
TNF‐α: tumor necrosis factor alpha

## 1 Introduction

Pain is a prevalent symptom in autoimmune diseases such as multiple sclerosis and rheumatoid arthritis, and its management remains a challenge. Based on the current understanding of their pathophysiological mechanisms, these diseases involve the dysregulated activation of the innate and adaptive immune system, leading to high levels of inflammation that damage tissue in the periphery and central nervous system along with the sensitisation of primary sensory neurons, culminating in pain and disability (Solaro, Trabucco, and Messmer 2013; Stephen 2001; Urits et al. 2019; Chisari et al. 2020; O'Connor et al. 2008).

Inflammation is not always detrimental, however. For instance, after injury, immune‐mediated inflammation can trigger regenerative mechanisms, as infiltrating macrophages assist in debris clearance or promote regeneration (Watanabe et al. 2019; Karin and Clevers 2016; Liu, Zhong, and Karin 2022). Inflammation is a dynamic process that can be initiated in response to various stimuli and then resolve when no longer required (Lo et al. 1999; Ji et al. 2011; Serhan, Chiang, and Van Dyke 2008). In individuals with multiple sclerosis (MS), an inflammatory autoimmune disease characterised by periods of attack and remission, these inflammatory processes can coincide with periods of disability. Although inflammation can resolve in MS, it is not always the case; persistent low‐grade inflammation from immune cells in nervous system tissue may lead to neurodegeneration, emphasising the importance of the resolution process (Vercellino et al. 2009; Centonze et al. 2010). Chronic inflammation can lead to disability and may also play a role in the induction of chronic pain. As inflammation is adaptive and designed to resolve, its persistence may indicate an impairment in the resolution process.

Dorsal root ganglion (DRG) neurons have an innate capacity for growth after injury to their distal axons (Mahar and Cavalli 2018; Holder and Clarke 1988). This plasticity can be demonstrated in cell culture. Dissociated DRG neurons can readily establish and send out axons in short periods of time, a process that can be encouraged by the priming of these cells with ‘conditioning lesions’ (Smith and Pate Skene 1997; Renthal et al. 2020; Mcquarrie and Grafstein 1973) or discouraged by inhibition of retrograde transport along the injured axon (Richardson and Verge 1986; Neumann and Woolf 1999). Furthermore, the structural plasticity of neurons can be modulated by pharmacological manipulation of ion channels. For example, inhibition of Ca^2+^ channel trafficking to the membrane (Patel and Dickenson 2016) with gabapentin can modulate synaptogenesis and neural circuitry rewiring (Fink et al. 2000; Takahashi, Jin, and Prince 2018). This effect is also observed in vivo, as gabapentinoids can be used to increase regeneration of axons after spinal cord injury (Sun et al. 2019; Tedeschi et al. 2022; Cragg et al. 2020). Similarly, Baclofen reduces the amplitude of voltage‐gated Ca^2+^‐currents in DRG neurons (Dolphin and Scott 1986; Hilton et al. 2022) and promotes their regeneration after spinal cord injury. The growth state of sensory neurons can also be modulated by inflammation (Benowitz and Popovich 2011; Richardson and Lu 1994; Steinmetz et al. 2005; Lu and Richardson 1991). Not only can the inflammatory response to injury, even potentially sterile injury, have effects on outgrowth, but inflammation triggered by adaptive or innate immunity can also affect outgrowth. Therefore, inflammation can be seen as a critical driver of structural plasticity and the growth status of sensory neurons (Leon et al. 2000).

It is also widely recognised that inflammation can alter the excitability of neurons, and traditional mechanisms of inflammation can result in increased action potential frequencies and lowered thresholds for action potential generation (i.e. sensitisation). Inflammatory mediators like tumor necrosis factor alpha (TNFα) and IL‐1β can regulate neuronal expression of voltage‐gated sodium and potassium channels, increasing neuronal excitability (Boettger et al. 2008; Schäfers and Sorkin 2008; Hakim et al. 2009; Richter et al. 2010; Khan et al. 2008). This phenomenon has been observed in both electrophysiological recordings of single cells (Lu et al. 2010) and nerve conduction studies (Eliav et al. 1999; Chacur et al. 2001). Heightened excitability and sensitisation of peripheral sensory neurons result in an increased perception of pain, although this phenomenon may resolve as inflammation subsides.

While inflammation can independently affect both the excitability and growth status of neurons, increasing excitability through electrical stimulation can promote axonal growth, indicating that there may be a connection between the two processes (Juckett et al. 2022; Senger et al. 2022, 2020). Additionally, some researchers have proposed that the inflammation‐induced changes in excitability may be directly involved in changes to neuronal growth status (Grubb and Burrone 2010; Webster et al. 2017; Hinman, Rasband, and Carmichael 2013). Increasing neuronal excitability using optogenetics enhances axonal growth in the presence of an inflammatory stimulus (Ecanow et al. 2022; Ward, Clanton, and English 2018). This suggests that the increase in neuronal excitability may be one of the mechanisms by which inflammation promotes structural plasticity.

Overall, the relationship between inflammation, excitability and the capacity for structural plasticity of DRG neurons is complex and likely involves multiple signalling cascades. To investigate this relationship, we conducted a series of experiments examining the effect of inflammation on neuronal excitability and neurite outgrowth. Our findings indicate that an early phase of increased neuronal excitability results in an increased growth status of primary sensory neurons, and that the neuron's excitability is intricately linked to its structural phenotype.

## 2 Materials and Methods

### 2.1 Animals

Unless otherwise stated, all mice are wild‐type C57/BL6, purchased from Charles River and included in experiments at 8 to 10 weeks of age (C57BL/6NCrl, RRID:IMSR_CRL:027), weighing approximately 20 g. A total of 60 mice were used. All mice were housed in ventilated cages at a maximum of 5 per cage, given access to food and water ad libitum and maintained in the University of Alberta facility under 12 h light/dark cycles under the Animal Ethics protocol AUP0000274.

### 2.2 Bone Marrow Derived Macrophage Cultures and Conditioned Media

Bone marrow‐derived macrophage cultures were generated under a previously described protocol (Friedman et al. 2023). Briefly, female and male 8‐ to 10‐week‐old C57BL/6 mice were euthanised by Dorminal (sodium pentobarbital, 1200 mg/kg) injected intraperitoneally. After euthanasia, bone marrow cells were isolated from femurs as single‐cell suspensions and filtered to remove excess debris. Bone marrow cells were allowed to differentiate into macrophages (Bone Marrow Derived Macrophage [BMDMs]) for 8 to 10 days in L929 media (Friedman et al. 2023). Upon completed differentiation, bone marrow‐derived macrophages were harvested and resuspended into freshly prepared Dulbecco's Modified Eagle's Medium (DMEM^−/−^) high glucose (4.5 g/L D‐Glucose, Gibco, Cat# 11960069) containing 1% each of sodium pyruvate (100 mM stock, Thermo Fisher, Cat# 11360070), Glutamax (100× stock, Thermo Fisher, Cat# 35050061), Pen/Strep (10 000 U/mL stock, Gibco, Cat# 15140–122) and FBS (Gibco, Cat# 12483020). This media is hereon referred to as ‘low‐serum media’. Cells were cultured in T75 flasks and were allowed to rest for 48 h after which a full change of media was performed. A small volume of TNFα (R&D Biosciences, Cat# 410‐MT, final concentration: 1 ng/mL) or vehicle (low‐serum media) was added and allowed to incubate for 24 h. After incubation, the stimulation media was fully replaced with a final volume of low‐serum media and the stimulated BMDMs were allowed to rest for 6 h. This ‘6‐h conditioned media (CM)’ was captured at the experimental endpoint, aliquoted and stored at −80°C until further usage avoiding freeze‐thaw cycles.

### 2.3 Dorsal Root Ganglia Neuron Cultures

Dorsal root ganglion (DRG) neurons were acquired from male and female mice using a modified protocol from previously described work (Maguire, Plemel, and Kerr 2023). Briefly, animals were euthanised by Dorminal (sodium pentobarbital) injected intraperitoneally. After injection, animals were monitored for level of consciousness, and dissections did not proceed until no response to toe pinch or corneal contact was observed. Cardiac punctures were performed to confirm euthanisation, and animals were perfused with 10 mL of ice‐cold saline. Perfused animals underwent spinal laminectomies and gross dissection of the spinal cord to expose the DRG. DRG were micro‐dissected from the spinal column, taking care to remove as much residual nerve as possible while avoiding damage to the DRG. Isolated DRG were placed in ice‐cold Hank's Balanced Salt Solution (HBSS^−/−^) until dissections were completed. To acquire single‐cell suspensions of DRG neurons, the HBSS^−/−^ was replaced with a warmed dilution of Stemxyme I (Worthington, Cat# LS004106) and DNase (Worthington, Cat# LS002007) in HBSS^−/−^ and incubated in a 37°C water bath for approximately 45 min. Following digestion, enzyme activity was quenched with equal volumes of low ovomucoid (Worthington, Cat# LS003086) and mechanically titrated with a P1000 pipette until tissue was fully dissociated. The cell suspension was filtered through a 70 um mesh filter (Biologix, Cat# 15–1070) and gently layered on top of a 20% bovine serum albumin (BSA) (Sigma Aldrich, Cat# A7906) solution. This layered gradient was spun at 300G for 10 min at room temperature to pellet neuronal cells and remove cellular debris (mainly myelin). Debris was gently removed, and the cell pellet was resuspended in a small volume of 0.5% BSA (Sigma Aldrich, Cat# A4161) in HBSS^−/−^ and quantified for neuronal yield with a haemocytometer. The cell suspension was adjusted to 1000 cells/100 μL and 100 μL of this suspension was added to equilibrated media in a 24 well (CellVis, Cat# P24‐1.5H‐N), poly‐D‐lysine‐coated plate (Sigma, Cat# P6407). For electrophysiological recordings, cells were plated onto glass coverslips (Fisher, Cat# 1254583) identically coated in poly‐D‐lysine and transferred into the electrophysiological recording setup at experimental timepoints. For experiments involving BMDM‐CM, the neuronal cell suspension was added directly into the equilibrated conditioned media. Unless otherwise stated, plated neurons were incubated at 37°C with 10% CO_2_ for 48 h. A full list of reagents used in cell culture experiments can be found in Table S1.

### 2.4 Pharmacology

For experiments involving the Kv7 channel agonist retigabine (RTG) and the CaMKII inhibitor KN93, a master stock was prepared by reconstituting lyophilised RTG (Tocris, Cat# 6233, 50 mM) or KN93 (Tocris, Cat# 1278, 2.5 mM) in dimethylsulfoxide (DMSO), aliquoting and storing at −20°C. On experimental timepoints, a 10x concentration was prepared by diluting thawed aliquots in DMEM. The final concentration of drugs was adjusted by direct dilution (1:10) into plate wells (50 and 100 μM final RTG, 500, 1000 and 2000 nM KN93).

### 2.5 Immunocytochemistry

At experimental endpoints, an equivalent volume of 8% paraformaldehyde (PFA) was added to the media of culture plates containing the adherent cells. Samples were incubated at room temperature in the diluted fixative for 15 min and then washed 3 times in Dulbecco's Phosphate Buffered Saline (DPBS). Permeabilisation and non‐specific IgG binding were blocked by 1‐h incubation with 10% normal donkey serum (Sigma, Cat# 566460) in DPBSTx 0.2% (DPBS with Triton‐X) at room temperature, followed by an overnight incubation with rabbit anti‐βIII tubulin (Sigma‐Aldrich, Cat# T2200, RRID:AB_262133) diluted in DPBS. On the following day, cells were washed 3 times in DPBS, incubated with DAPI (Invitrogen, D1306) and 594‐conjugated anti‐rabbit secondary antibodies (Jackson ImmunoResearch Labs Cat# 711‐586‐152, RRID:AB_2340622) for 1 h at room temperature before a final three washes. Imaging was performed on an ImageXpress Micro system and analysed using MetaXpress 6.

### 2.6 Neurite Extension Well Average Analysis

Imaging was performed on an ImageXpress Micro system, using a single protocol for all experiments. Fluorescence images were collected with a 10× objective, corresponding to a field‐of‐view of 1406 μm^2^ per image, tiled in a 6‐by‐6 grid with 10% overlap. Basic neurite extension was performed using the ‘Neurite Extension’ plugin in the MetaXpress 6 software. Briefly, images were skeletonised based on a consistent threshold of βIII tubulin fluorescence immunoreactivity to quantify total outgrowth. Data was aggregated by well, averaging outgrowth of Total # of Neuronal Cell Bodies/Total # of Neurites. Experiments were replicated with internal controls for normalising, where one replicate corresponds to a single well's average for that condition. Additional metrics were retrieved from the ‘Neurite Extension’ analysis, including # of Neuronal Cells, Total Outgrowth, # of Branches per Cell, # of Processes per Cell and % Significant Outgrowth (data not shown).

### 2.7 Whole‐Cell Current Clamp Electrophysiology

Action potential recordings from mouse DRG neurons were acquired in current‐clamp mode using an Axopatch 200B amplifier (Molecular Devices), a Digidata 1440 digitizer and Clampex10 software (Molecular Devices). Whole‐cell configuration was obtained in voltage‐clamp mode before manually switching to current‐clamp recording mode. Recordings were filtered at 5 kHz and sampled at 10 kHz. Patch pipettes were manufactured from soda lime capillary glass (Thermo Fisher Scientific) using a Sutter P‐97 (Sutter Instrument) puller. Electrodes had a tip resistance of 2–4 MΩ when filled with an internal (pipette) solution. Pipette solution was comprised of 130 mM K‐gluconate, 4 mM Mg‐ATP, 0.3 mM Na‐GTP, 10 mM EGTA, 2 mM CaCl2 and 10 mM HEPES (adjusted to pH 7.2 with KOH). The bath was perfused with an external solution containing 135 mM NaCl, 5 mM KCl, 1 mM CaCl2, 1 mM MgCl2 and 10 mM HEPES (adjusted to pH 7.3 with NaOH). Patch clamp experiments were performed at room temperature (22 ± 1°C). DRG neuron dimensions (cell size) were estimated using a microscope eyepiece reticle (27 mm, 10 mm scale). For identification of IB4+ cells, DRG neurons were labelled with isolectin GS‐B4 Alexa Fluor 488‐conjugated antibody (Thermo Fisher, Cat# I21411) at least 15 min prior to transferring cells to the recording chamber. Images of DRG neurons in the recording chamber were acquired using a high‐resolution USB2.0 CMOS, 1280 × 1024, Camera (Thorlabs, DCC1645C) and ThorCam software. The resting membrane potential was determined immediately following whole‐cell break‐in at *I* = 0 pA. Cells that were unable to maintain a giga‐ohm seal and with no resting membrane potential upon whole‐cell break were deemed unviable and were not recorded from. Threshold (Rheobase) was established by the first action potential to be elicited by a series of 3 s stepwise current injections that increased from 0 pA in 10 pA increments. Action potential frequencies were calculated by the number of spikes during the 3 s stepwise current injections from 0 pA in 10 pA increments. The frequency of action potentials during acute retigabine application (RTG) was analysed using the event detection, threshold search feature of Clampfit 10.7. The frequency of action potentials was calculated by the number of spikes over the time exposed to a specific condition (control, RTG application, washout). The baseline was set at the resting membrane potential at the beginning of the recording. The threshold level for the inclusion criteria of an action potential was set at 0 mV. Patch clamp recordings and analysis were performed independent from DRG extraction and culture; as such, the electrophysiologist was blinded to all experimental groups.

### 2.8 Experimental Autoimmune Encephalomyelitis Induction and Behavioural Assessment

Experimental autoimmune encephalomyelitis (EAE) was induced in female C57BL/6 mice (*n* = 12; 8–10 weeks old; Charles River) by subcutaneously injecting 50 μg of myelin oligodendrocyte glycoprotein (MOG_35‐55_) emulsified in complete Freund's adjuvant (CFA) (Hooke Laboratories, Hook Kits, Cat# EK‐2110). Mice were given 100 ng of pertussis toxin, *Bordetella pertussis* (Hooke Laboratories, Cat# BT‐0105) via IP injection on the day of induction and 24 h later. Mice were monitored daily until the end of the experiment.

Mice were assessed for pain hypersensitivity by measuring withdrawal thresholds to punctate mechanical stimulation using calibrated von Frey Hair monofilaments. Mechanical withdrawal thresholds were measured by the von Frey assay. Animals were repeatedly habituated to a 7.5 × 10 × 7.5 cm plexiglass box on a suspended mesh platform and allowed to explore for 30 min. On test days, the up‐down method was used to determine the 50% withdrawal threshold (Dixon 1991). Briefly, filaments of various forces (0.04–4 g) were applied to both hind paws and positive and negative responses were recorded. The 50% withdrawal threshold was calculated, as previously described (Chaplan et al. 1994). Prior to immunisation, baseline withdrawal thresholds were recorded. Mice were then tested on days 7, 11 and 15 after immunisation for EAE. Mice were treated with either RTG (10 mg/kg, IP, *n* = 6) or vehicle (DMSO in saline, 10%, *n* = 6) daily beginning on day 7 (after von Frey testing had been completed). DRGs from these mice were harvested on day 16 and subjected to electrophysiological analysis using the same parameters described above. All animal experiments were performed according to the Canadian Council on Animal Care's Guidelines and Policies with approval from the University of Alberta Health Sciences Animal Care and Use Committee.

### 2.9 Statistical Analyses

No formal sample size calculations were performed for this study due to limitations on the number of replicates feasible for each experiment. Sample sizes were determined based on standards commonly used in the field for similar experimental designs. Where applicable, experiments included equal numbers of male and female subjects, and no mixed‐sex samples were used in analyses involving both sexes. Inclusion and exclusion criteria were minimal, with no animals excluded from any experiments.

Statistical analyses were conducted using PRISM v10. For comparisons involving more than two groups with a single variable, one‐way ANOVA was used. For experiments involving two variables, such as sex and treatment, two‐way ANOVA was performed. In all other cases, two‐group comparisons were conducted using *t*‐tests. No formal assessments for normality or outliers were carried out on any datasets.

## 3 Results

### 3.1 DRG Neuronal Plasticity After Stimulation With BMDM Conditioned Media

**FIGURE 1:**
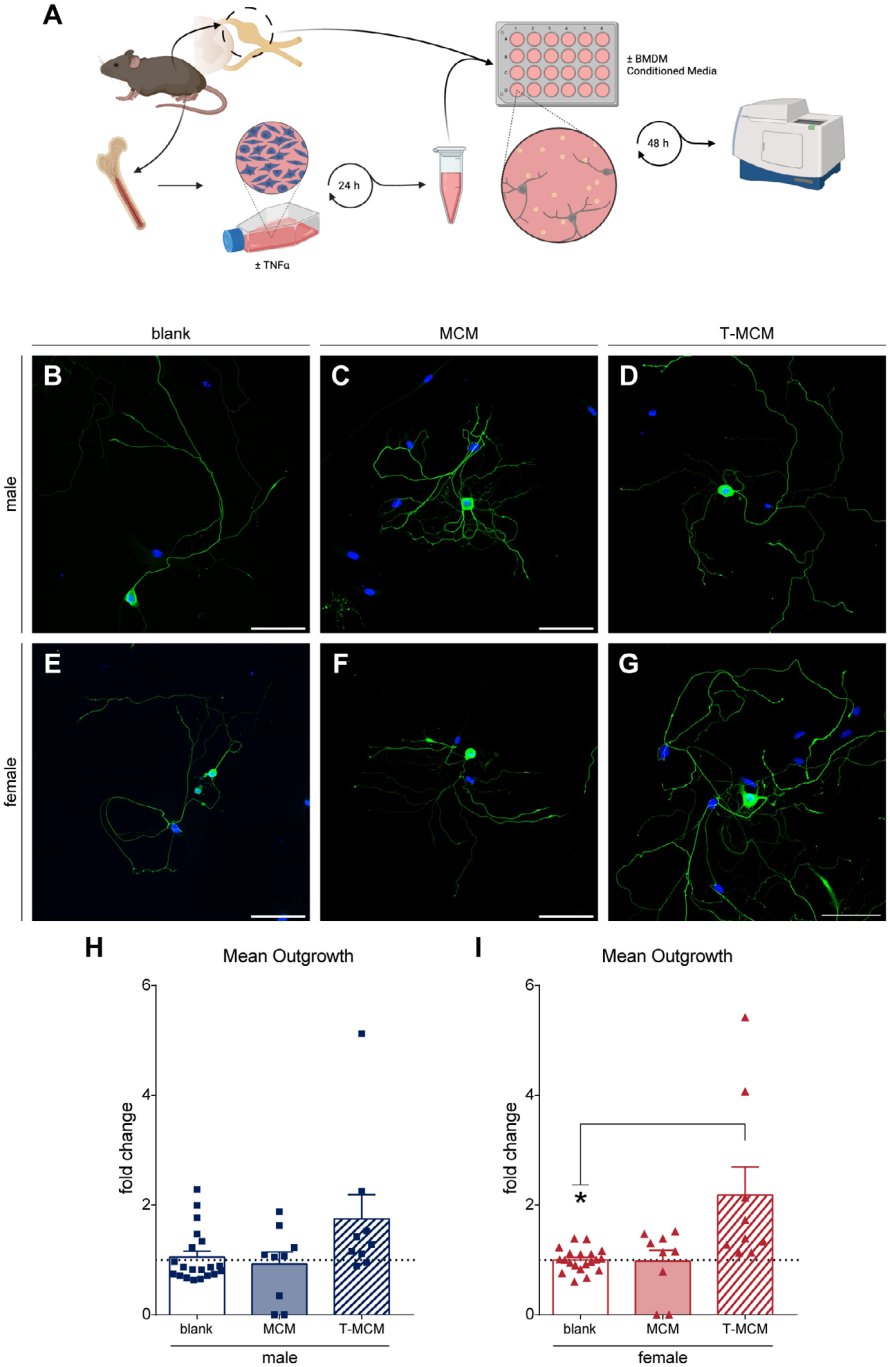
Male and female DRG neurons display increased neurite outgrowth in anti‐inflammatory and female‐specific pro‐inflammatory BMDM‐conditioned media. (A) Schematic of DRG neuron cultures and CM stimulation. (B–G) Representative images of neurites stained with βIII tubulin at the experimental endpoint. Quantification of male (H) and female (I) neurites with sex‐matched condition media treatments, normalised to the extent of outgrowth measured in the control (blank) conditions. Scale bar = 50 μm; *p < 0.05. Data is analysed by one‐way ANOVA with Dunnett's multiple comparison test. Bar graphs represent mean ± SEM, with *n* = 9–21 wells from three independent cell culture preparations.

Previous experiments from our group have shown that the conditioned media from male and female macrophages after pro‐inflammatory stimulation elicit different pain phenotypes when injected into the hind paw of adult mice (Friedman et al. 2023). Male mice were relatively resistant to change in their tactile thresholds after injection of macrophage‐conditioned media while females exhibited heightened pain sensitivity (Friedman et al. 2023). In the current study, we sought to establish the relationship between the growth status of sensory neurons and the conditioned media that elicited these different pain phenotypes. We first cultured DRG neurons in the base media used in macrophage‐conditioned media experiments to confirm if neurons would readily survive and extend neurites in media not specifically formulated for neuronal culture. Optimisation experiments confirmed that DRG neurons attached and began neurite outgrowth within 24 h post‐plating in base, ‘low‐serum’ media and survived at least 96 h in culture (data not shown). We next investigated the effect on the outgrowth of neurons in the presence of conditioned media from unstimulated (MCM) or TNFα‐stimulated (T‐MCM) male or female bone marrow‐derived macrophages. Neurons were cultured for 48 h in the presence of these different conditioned media, and structural plasticity was assessed by the pan‐neuronal and cytoskeletal βIII tubulin staining (Figure 1B–G). Mean outgrowth of neurites from neurons was quantified at the experimental endpoint for both male (Figure 1H) and female (Figure 1I) treated neurons. Both male and female neurons incubated with the standard, unstimulated macrophage condition media (MCM) had no change in outgrowth compared to regular media‐treated controls. However, female DRG neurons specifically demonstrated an increase in neurite outgrowth after incubation with female T‐MCM (1‐way ANOVA, *F*_2,35_ = 7.635, *p* = 0.0018, Figure 1I). Male neurons incubated with the male equivalent T‐MCM did not exhibit a statistically significant increase in neurite outgrowth (1‐way ANOVA, *F*_2,35_ = 3.035, *p* = 0.0609, Figure 1H).

### 3.2 Electrophysiological Assessment of DRG Neurons Early After Culturing

**FIGURE 2:**
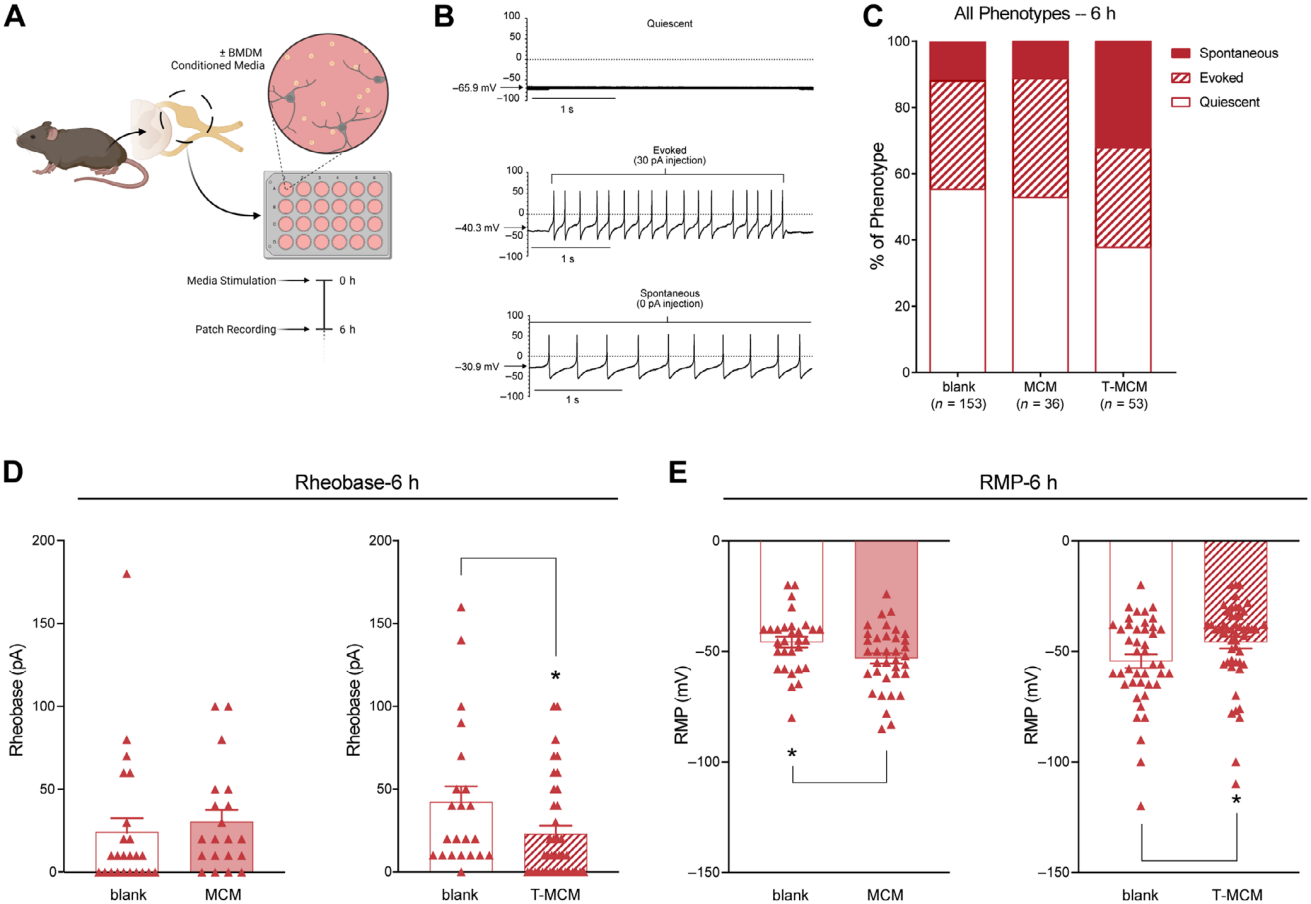
Whole‐cell patch clamp recordings of female DRG neurons incubated in BMDM‐conditioned media (CM) for 6 h reveal distinct firing patterns and electrophysiological profiles. (A) Schematic of experimental workflow; neurons were recorded 6 h post‐plating and CM stimulation. (B) Example traces of the three characteristic firing patterns: Quiescent, Evoked and Spontaneous. (C) Quantification of categorical labels for stereotypic firing patterns across CM treatments. Quantification of rheobase (D), resting membrane potential (RMP) (E) across CM treatments. *p < 0.05. Data is analysed by unpaired two‐tailed *t*‐test. Bar graphs represent mean ± SEM, with *n* = 20–51 cell recordings per group from three independent cell culture preparations. Categorical data is represented by parts‐of‐whole transformation and analysed by Fisher's exact test on untransformed values.

The function of neurons is dictated by their structure and excitability. We next assessed the excitability profiles of neurons treated with the different macrophage‐conditioned media using whole‐cell current clamp recordings, incubated under identical conditions to previous experiments (Yousuf et al. 2020). We decided to focus on female DRG neurons due to previous work indicating a specific mechanism of pain in our autoimmune inflammatory mouse model (Yousuf et al. 2019; Mifflin et al. 2019; Friedman et al. 2019) as well as the female bias of autoimmunity and chronic pain in the human population (Vos et al. 2015). We analysed general metrics of neuronal excitability, including the minimum electric current required to elicit an action potential (rheobase) and the resting membrane potential (RMP) after 6 h of incubation in the different conditioned media. We found that all conditions displayed varying degrees of spontaneous activity and quiescence, with the T‐MCM‐treated neurons exhibiting the most spontaneous activity (Figure 2C). While neurons treated with MCM had more negative RMP compared to the media‐only control (two‐tailed *t*‐test, MCM RMP: *t* = 2.156, df = 64, *p* < 0.05, Figure 2E), the rheobase of these MCM‐treated neurons was unchanged (Figure 2D). Neurons treated with T‐MCM, however, exhibited a lower rheobase and had less negative resting membrane potential (RMP) relative to the media‐only treated neurons (two‐tailed *t*‐test, T‐MCM Rheobase: *t* = 2.019, df = 59, *p* < 0.05; T‐MCM RMP: *t* = 2.115, df = 92, *p* < 0.05, Figure 2D,E). Interestingly, the T‐MCM‐treated neurons that had a higher excitability profile overall (i.e. most spontaneous activity, lowered rheobase and less negative resting membrane potential) at 6 h, correlated with the neurons exhibiting the greatest structural outgrowth at 48 h described in Figure 1. These findings suggest a potential positive relationship between increased early excitability of the sensory neuron and later increased structural plasticity.

### 3.3 Electrophysiological Assessment of DRG Neurons in Established Cultures

**FIGURE 3:**
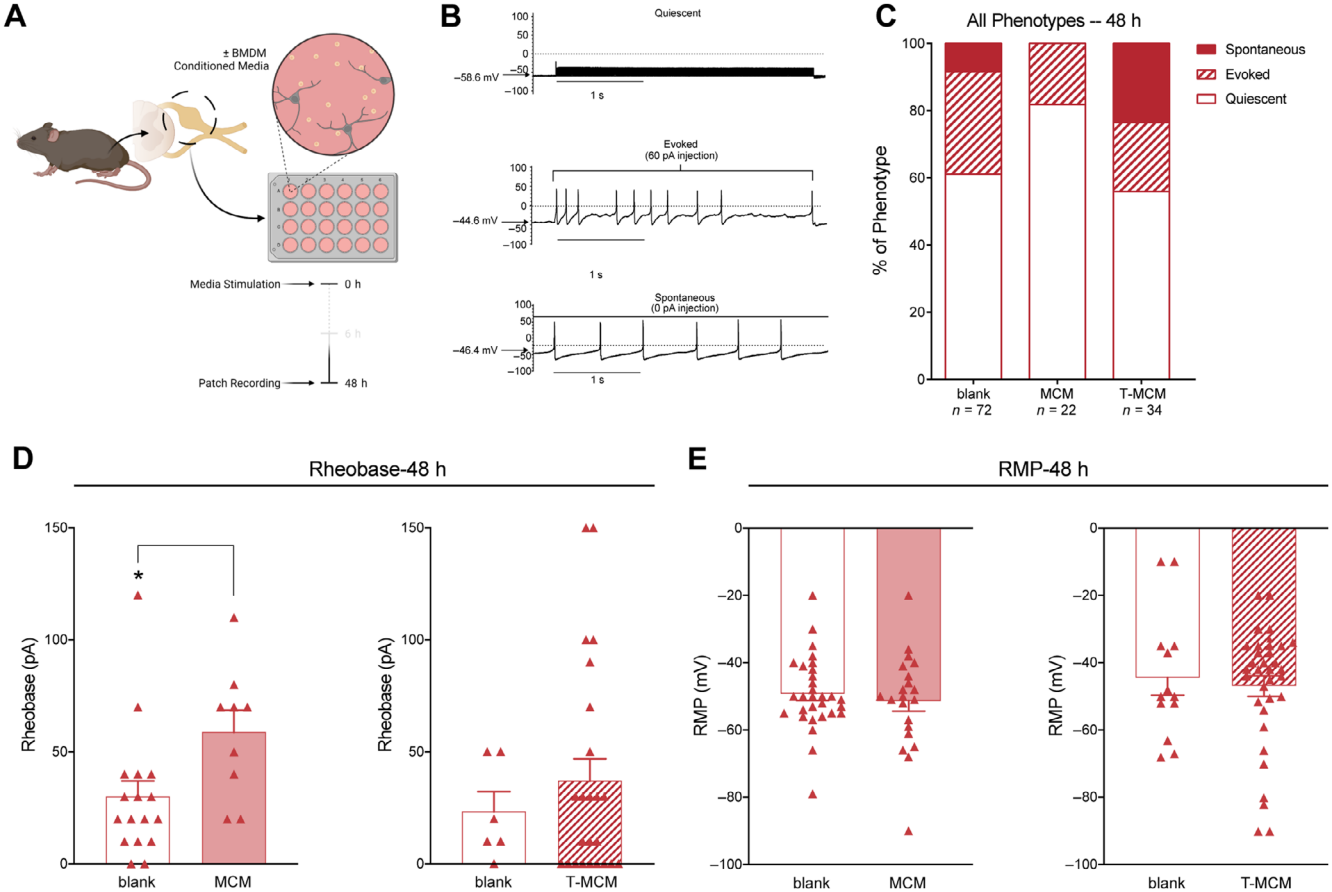
Whole‐cell patch clamp recordings of established female DRG neurons incubated in BMDM‐conditioned media (CM) reveal distinct firing patterns and electrophysiological profiles. (A) Schematic of experimental workflow; neurons were recorded 48 h post‐plating and CM stimulation. (B) Example traces of the three characteristic firing patterns: Quiescent, Evoked and Spontaneous. (C) Quantification of categorical labels for stereotypic firing patterns across CM treatments. Quantification of rheobase (D) and resting membrane potential (RMP) (E) across CM treatments. *p < 0.05. Data is analysed by unpaired two‐tailed *t*‐test. Bar graphs represent mean ± SEM, with *n* = 22–34 cell recordings per group from three independent cell culture preparations. Categorical data is represented by parts‐of‐whole transformation.

To evaluate neurons at later time points in culture, we conducted an identical electrophysiological assessment 48 h after plating. Notably, we observed an overall shift in the excitability profiles in all treatment conditions. Specifically, the MCM condition exhibited no spontaneous activity at this later timepoint, also exhibiting an elevated rheobase and the most negative resting membrane potential (two‐tailed *t*‐test, MCM Rheobase: *t* = 2.410, df = 24, *p* < 0.05; MCM RMP: *t* = 0.639, df = 50, *p* = 0.53, Figure 3C–E). In contrast, the T‐MCM‐treated neurons still displayed some spontaneous activity but were identical in rheobase and resting membrane potential compared to their media‐only controls (Figure 3C–E).

### 3.4 Growth Status and Electrophysiological Characteristics of DRG Neurons

**FIGURE 4:**
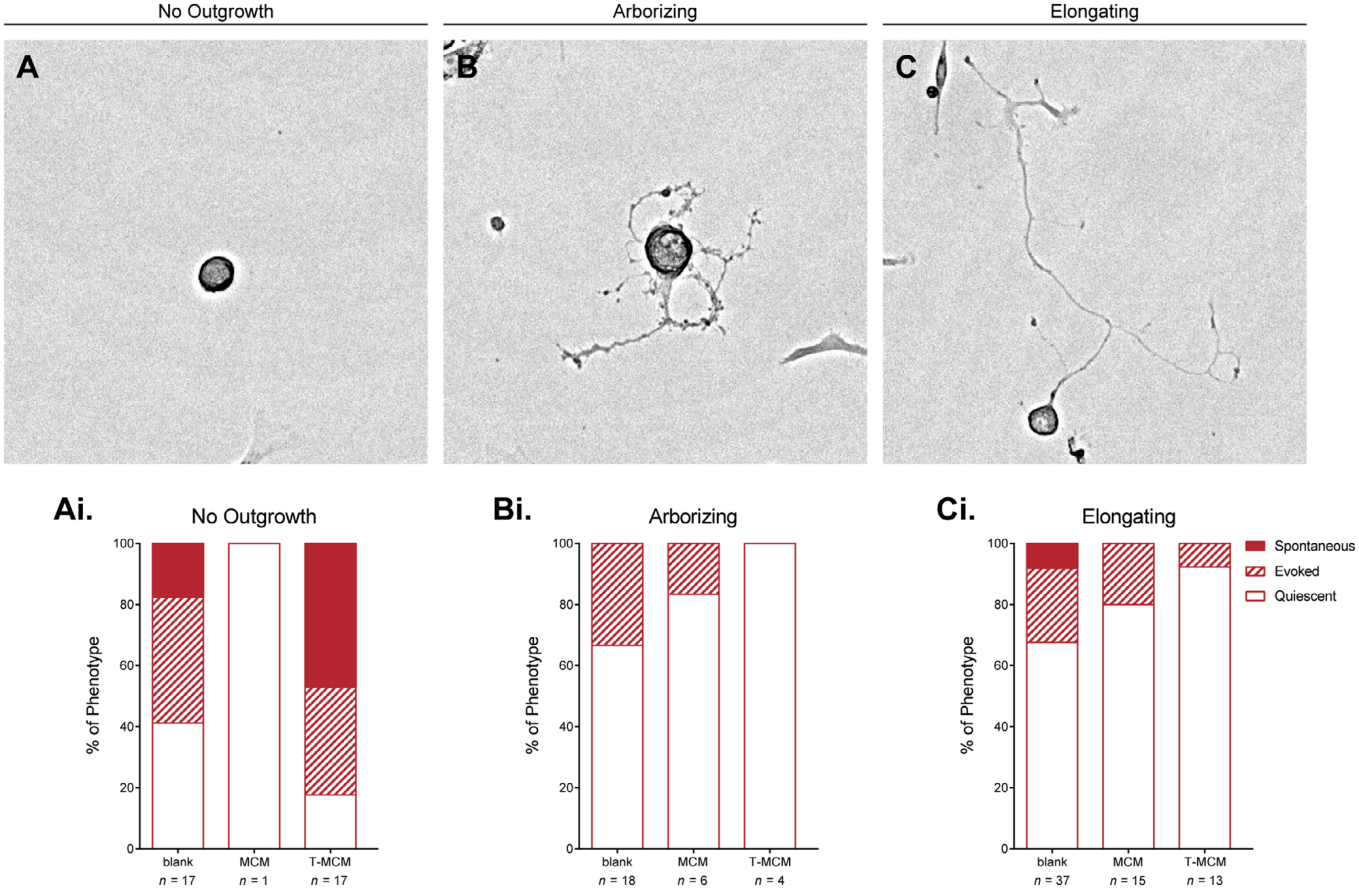
Whole‐cell patch clamp recordings of established female DRG neurons with distinct structural phenotypes have distinct firing patterns and electrophysiological profiles. (A–C) Representative brightfield images of the distinct neuronal phenotypes (*no outgrowth*, *arborizing*, *elongating*) observed at 48 h post‐plating and CM stimulation. (Ai–Ci) Quantification of categorical labels for stereotypic firing patterns across CM treatments. Categorical data is represented by parts‐of‐whole transformation.

As we were interested in relating electrical excitability to structural plasticity, we noted that neurons adopt distinct morphologies 48 h after plating, consistent with observations from Figure 1. At this time point, neurons were consistently observed to fall into three categories: a population of neurons yet to extend any neurites (*no outgrowth*); a population that display a highly complex, ‘arborizing’ phenotype characterised by numerous branches from the primary outgrowth (*arborizing*) and a population that extend ‘elongating’ neurites characterised by low amounts of branching and a high displacement of primary growth (*elongating*) (Figure 4A–C). Identifying and subdividing these morphological populations by visual inspection at the time of patch clamp recording, we found that the spontaneous activity seen in the T‐MCM condition was restricted to neurons with no outgrowth (Figure 4Ai). Furthermore, neurons in the ‘arborizing’ and ‘elongating’ categories were primarily quiescent regardless of treatment condition (Figure 4Bi,Ci). Overall, these data suggest that incubation of neurons with different types of inflammatory‐conditioned media conveys unique excitability profiles in neurons that have yet to display outgrowth and that this can inform the extent of outgrowth.

### 3.5 Structural Plasticity Is Modulated by Kv7 Channel Activity

**FIGURE 5:**
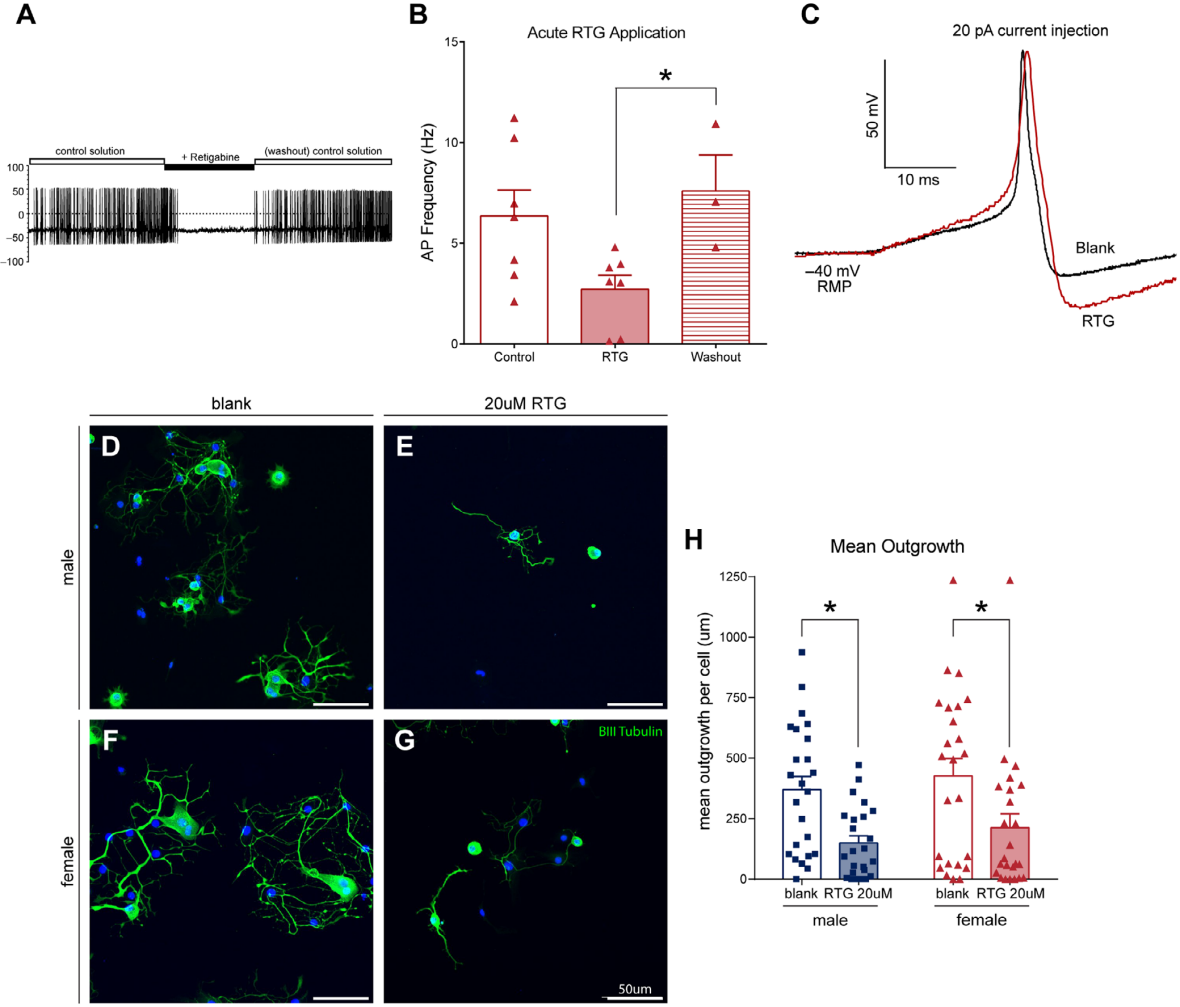
Neurite outgrowth of DRG neurons is diminished by retigabine (RTG) treatment. (A) Example traces of spontaneously firing DRG neurons that become quiescent under RTG treatment. (B) Quantification of action potential (AP) frequency in binned time domains centred around the period of RTG stimulation. (C) Individual traces of unstimulated and RTG‐stimulated APs. Note the deeper AHP curve and slower return to RMP in the RTG trace. (D–G) Representative images of neurites stained with βIII tubulin at the experimental endpoint after treatment with blank media (D, F) or RTG (E, G). (H) Quantification of male and female neurites after treatment with RTG or control (blank media). Scale bar = 50 μm; *p < 0.05. Data is analysed by an unpaired two‐tailed *t*‐test. Bar graphs represent mean ± SEM with *n* = 24 wells from three independent cell culture preparations. Categorical data is represented by parts‐of‐whole transformation.

To assess the direct impact that electrical activity/excitability has on structural plasticity, we turned to pharmacological manipulation of these neurons in our in vitro system. Retigabine (RTG) is an anticonvulsant drug that was initially developed as a treatment for epilepsy. It acts as a positive allosteric modulator of voltage‐gated potassium channels (Kv7), which stabilises the resting membrane potential and reduces neuronal excitability (Yekkirala et al. 2017; Brown and Passmore 2009; Kim et al. 2015). We repurposed this drug to act as a general inhibitor of neuronal activity to mimic the quiescent phenotype we identified in vitro. The effect of RTG is near instantaneous, as repetitively evocable neurons exhibit a complete loss of electrical activity upon wash‐in of RTG during recording (Figure 5A,B). Repetitive firing of neurons is then restored when RTG is washed off (1‐way ANOVA, *F*_2,14_ = 4.40, *p* < 0.05, Control vs. RTG, *p* = 0.0584, RTG vs. Washout, *p* < 0.05, Figure 5B). Basal neurite outgrowth of both male and female DRG neurons cultured in the presence of retigabine is also significantly reduced after 48 h in culture (2‐way ANOVA, treatment effect *F*_1,92_ = 15.54, *p* < 0.05, Figure 5H). Although the quiescent phenotype was primarily shown in the later stages of outgrowth, we additionally tested the addition of RTG at a later timepoint (24 h post plating), but this had an identical effect to the early addition (data not shown). These data indicate that neurons are dependent on some level of spontaneous activity for axonal outgrowth.

### 3.6 Plasticity Driven by BMDM Conditioned Media Is Dependent on Excitability

**FIGURE 6:**
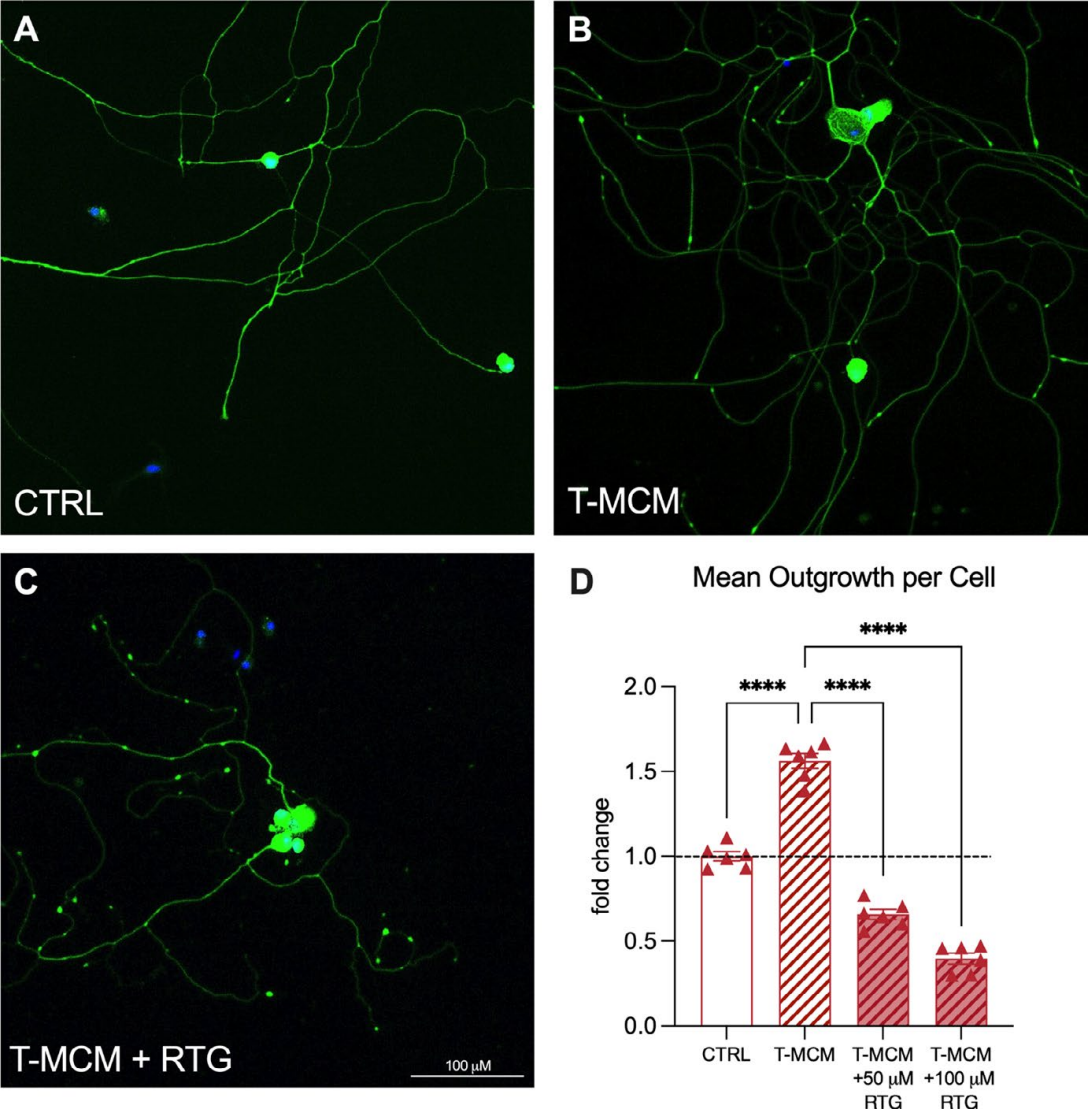
Retigabine prevents the growth‐promoting action of inflammatory conditioned media. (A–C) Representative images of neurites from mouse DRG neurons after culture for 48 h with either no treatment, BMDM‐conditioned media treatment, or BMDM‐conditioned media +100 μM RTG treatment. (D) Quantification of mean area of neurite outgrowth. Scale bar = 100 μm, and dashed line represents mean of untreated control; ****p < 0.0001. Data is analysed by one‐way ANOVA. Bar graphs represent mean ± SEM normalised to untreated controls, with *n* = 6 wells from one cell culture preparation, and the dashed line represents the mean of the untreated control bar.

To assess whether the neurite growth‐promoting effects of T‐MCM are dependent on changes to the excitability profile of the neurons, we treated DRG neurons for 48 h with T‐MCM in the presence or absence of RTG. As seen previously (see Figure 1), compared to neurons treated with media only, T‐MCM promoted a significant increase in mean outgrowth (Figure 6A,B,D). This effect was abrogated by co‐incubation of T‐MCM with RTG (1‐way ANOVA, treatment effect *F*_2,33_ = 194, *p* < 0.0001, Figure 6C,D).

### 3.7 Increased Excitability Mediates Plasticity Through CaMKII

**FIGURE 7:**
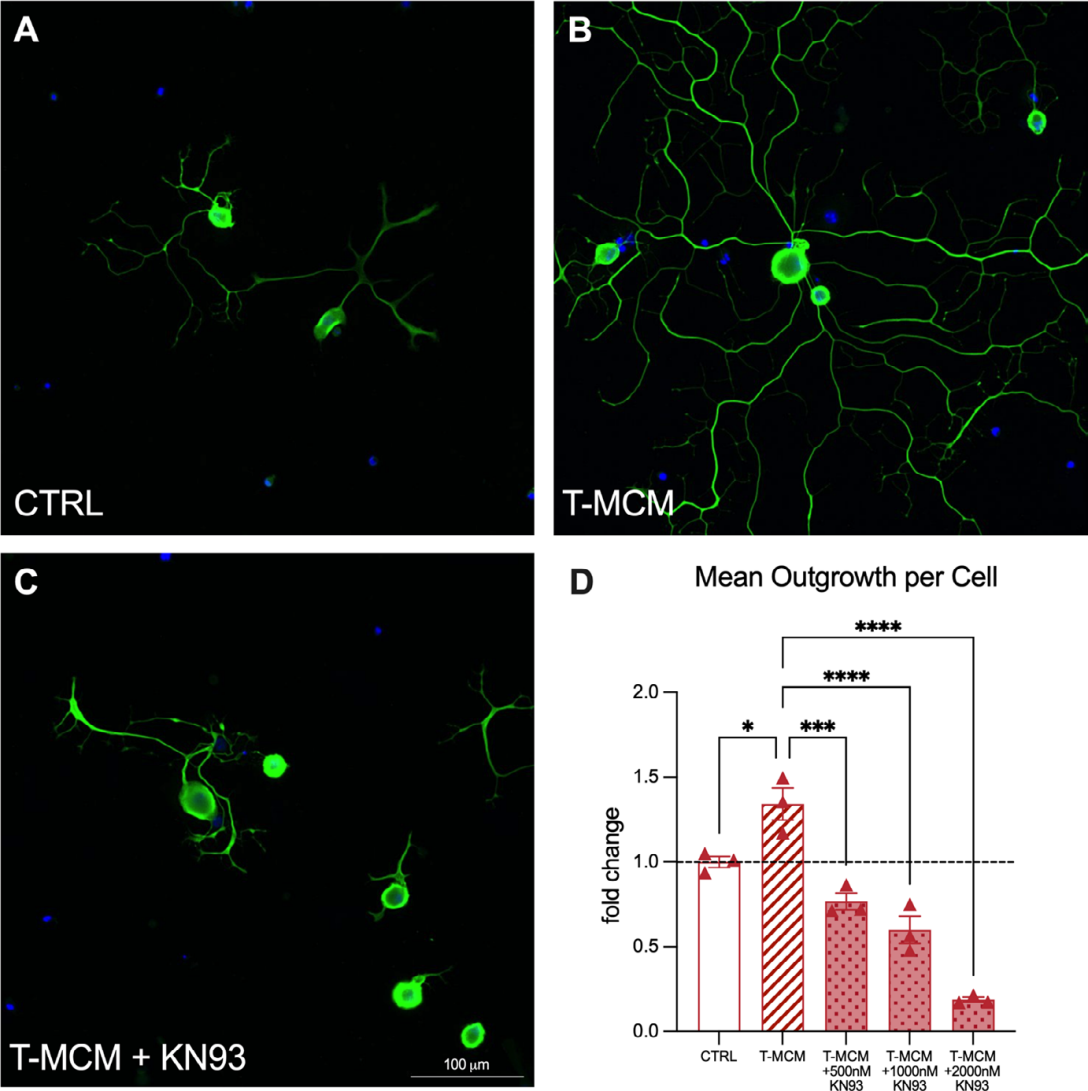
KN93 prevents the growth promoting action of inflammatory conditioned media. (A–C) Representative images of neurites from mouse DRG neurons after culture for 48 h with either no treatment, BMDM‐conditioned media treatment, or BMDM‐conditioned media +500 nM KN93 treatment. (D) Quantification of mean area of neurite outgrowth. Scale bar = 100 μm, and dashed line represents mean of untreated control; *p < 0.05, ***p < 0.001, ****p < 0.0001. Data is analysed by one‐way ANOVA. Bar graphs represent mean ± SEM normalised to untreated controls, with *n* = 3 wells across two independent cell culture preparations, and dashed line represents mean of untreated control bar.

To determine the downstream signals that mediate the increased outgrowth in response to T‐MCM and the concomitant increase in neural activity, we next examined the effects of blocking CaMKII, an established mediator of neuronal growth. We treated DRG neurons for 48 h with T‐MCM in the presence or absence of the CaMKII inhibitor KN93. As expected, neurons treated with T‐MCM exhibited increased outgrowth (Figure 7A,B). Much like the effects of blocking neural activity with RTG, treating neurons with KN93 in the presence of T‐MCM significantly attenuated this enhanced growth (1‐way ANOVA, treatment effect *F*_2,6_ = 20.19, *p* = 0.0022, Figure 7C,D).

### 3.8 Attenuating DRG Excitability Prevents Pain in Mice With EAE

**FIGURE 8:**
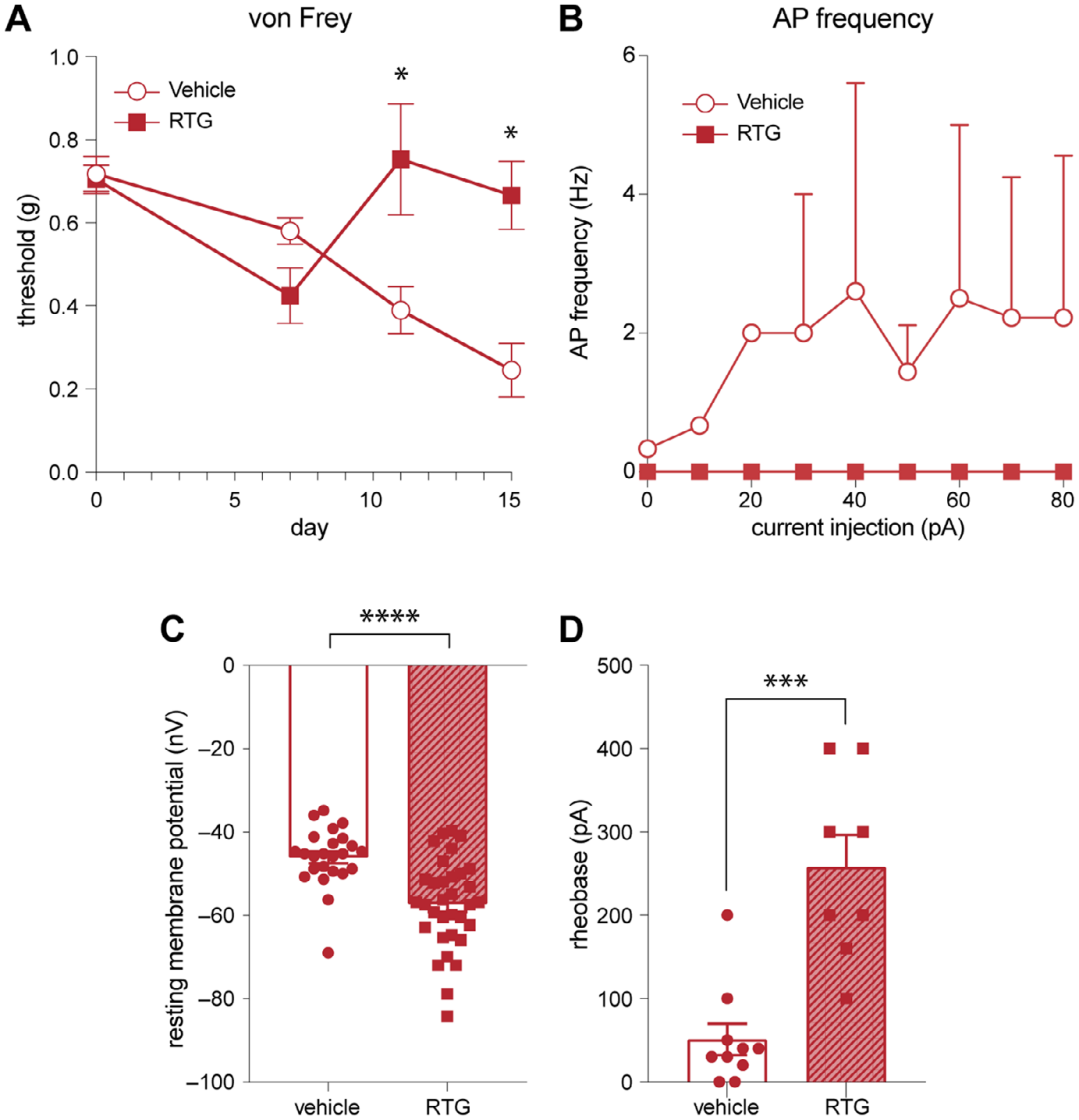
Retigabine prevents pain hypersensitivity in the EAE model of neuroinflammatory disease. (A) Withdrawal thresholds to von Frey hair stimulation in mice immunised for EAE and treated with vehicle control (left) or RTG (10 mg/kg) (right). Vehicle‐treated mice exhibit stereotypical reductions in withdrawal thresholds indicative of pain hypersensitivity. Mice that begin treatment with RTG on day 7 post‐immunisation exhibit a complete reversal of these behaviours. *p < 0.05, two‐way ANOVA, Tukey host hoc test. (B) Number of action potentials from DRGNs of EAE‐immunised mice treated with retigabine (RTG, 100 μM) versus vehicle control. Frequency of action potentials (Hz) was recorded from 3 s current injections in 10 pA increments. (C) Resting membrane potential (RMP, mV) from DRGNs of EAE‐immunised mice treated with retigabine (RTG, 100 μM) versus vehicle control. ****p < 0.001, two‐tailed unpaired Student's *t*‐test, DRGNs treated with RTG (100 μM) versus vehicle control. (D) Action potential injection threshold (rheobase, pA) from DRGNs of EAE‐immunised mice treated with retigabine (RTG, 100 μM) versus vehicle control. Line graphs represent mean ± SEM, with *n* = 8 animals from one in vivo experiment. Bar graphs represent mean ± SEM, with *n* = 24–33 cell recordings from one cell culture preparation. ***p < 0.001, two‐tailed unpaired Student's *t*‐test, DRGNs treated with RTG (100 μM) versus vehicle control.

Given the robust effects on neural activity and outgrowth from DRG neurons treated with RTG in the presence of inflammatory macrophage‐conditioned media, we next wanted to determine how this might impact pain in the context of neuroinflammatory disease. We have previously described the emergence of pain hypersensitivity to tactile stimulation in the hind paws of mice with experimental autoimmune encephalomyelitis (EAE) that is accompanied by significant increases in DRG neuron excitability and activity (Yousuf et al. 2019). Mice were immunised for EAE and 7 days after immunisation we began treatment with either RTG (10 mg/kg, IP) or vehicle control. Vehicle‐treated mice exhibited well‐characterised, significant reductions in paw withdrawal thresholds indicative of pain hypersensitivity that became statistically different from baseline values on day 11 post‐immunisation. In contrast, mice that began treatment with RTG on day 7 displayed a complete reversal of this behavioural hypersensitivity (Figure 8A, two‐way RM ANOVA, treatment effect *F*_1,10_ = 6.035, *p* < 0.05). RTG treatment effectively supressed action potential firing from DRG nociceptors compared to vehicle‐treated mice (two‐tailed *t*‐test, *t* = 6.660, df = 16, *p* < 0.05, Figure 8B) and altered the overall excitability of these neurons by lowering the resting membrane potential and significantly elevating the rheobase (two‐tailed *t*‐test, RTG RMP: *t* = 4.440, df = 16, *p* < 0.05; RTG Rheobase: *t* = 5.090, df = 16, *p* < 0.05, Figure 8C,D). Collectively, reducing DRG excitability with RTG limits DRG axon outgrowth in vitro and pain hypersensitivity following EAE in vivo.

## 4 Discussion

### 4.1 BMDM Conditioned Media Conveys Plastic Potential to DRG Neurons

Building upon previous research indicating sex differences in innate immune inflammatory activity, we have investigated the sex‐specific changes in DRG sensory neuron structural plasticity and excitability when exposed to inflammatory mediators from innate immune cells (BMDMs). Here, we demonstrate that incubating peripheral neurons with sex‐matched inflammatory conditioned media from innate immune cells can impact both the structural plasticity and the excitability profile of these neurons. We find that excitability parameters of neurons that exhibit high amounts of structural outgrowth correspond to the greatest shift in excitability, becoming quiescent after an initial period of high spontaneous activity. Furthermore, we demonstrate that a pharmacological intervention limiting neuronal activation (RTG/KN93, Figures 6 and 7) can prevent this capacity for structural plasticity in the inflammatory conditions. However, as both RTG and KN93 independently reduced neurite outgrowth below vehicle control levels, further investigation is required to determine whether these molecules operate through distinct pathways from or intersecting with the pathway influenced by T‐MCM.

The lack of effect observed from conditioned media derived from untreated macrophages was surprising, as previous literature has reported growth‐promoting effects of conditioned media even from unstimulated macrophages. The condition effect may be influenced by several variables, including species differences, macrophage sources and culture paradigms. These variations can influence macrophage activation states beyond direct cytokine stimulation. In this study, we ensured that macrophage cultures were allowed to return to a basal resting state before the stimulation paradigm. However, it remains unclear whether the lack of effect from the unstimulated CM is due to the absence of a conditioning influence or if the effect is simply too subtle to be detected with the current level of statistical power.

It is important to note that TNFα and the associated T‐MCM used in this study represent just one type of inflammatory environment, specifically modelling autoimmune‐related inflammation. While other molecules, such as LPS, are commonly used to model inflammation arising from infection, our focus was to investigate pathways relevant to autoimmune conditions. Future studies will be required to determine whether the plasticity changes we observed are conserved across other inflammatory models, such as LPS‐driven inflammation or other equivalents.

### 4.2 DRG Neurons Are Highly Plastic Cells

In early neuronal development, the maturation of neurons involves complex signalling cascades that are crucial for the transcriptional profile and development of the functional component of these cells (Ernsberger 2009; Yoshikawa et al. 2013; Raible and Ungos 2006). Maintenance of neuronal circuitry in adult organisms is tightly regulated, and these plastic processes can result in adaptive or maladaptive consequences depending on the context. For instance, heightened pain sensitivity during inflammation may trigger acute plasticity that is adaptive for preventing further injury, but intense or prolonged inflammation also has the capacity to promote signalling within sensory neurons that leads to long‐term changes and chronic pain syndromes (Khomula et al. 2021; Joseph and Levine 2010; Kandasamy and Price 2015).

Pain management is a critical aspect of first‐line treatments for nervous system injury. However, strategies that directly address pain without considering the structural consequences have proven to be ineffective in preventing chronic pain. Thus, it may be worthwhile to consider both the functional and structural aspects of neural plasticity when developing pain management strategies. This involves understanding the interplay between activity‐dependent phenomena and regenerative outgrowth and investigating the mechanisms that link neuronal excitability and structural plasticity. Adopting such an approach could lead to more effective treatments for chronic pain syndromes. Emerging from current paradigms that tend to view heightened neuronal excitability following injury as detrimental due to the risk of excitotoxicity (Dong, Wang, and Qin 2009; Hoffe and Holahan 2022), the current study supports an alternative perspective: the necessity of preserving an early phase of excitability to facilitate proper neural regeneration. While efforts to curb excessive excitability post‐injury are well‐founded, these interventions might inadvertently hinder the inherent pro‐regenerative processes associated with the initial surge in neuronal excitability and activity. By excessively dampening this early phase, there could be an unintended loss of the favourable conditions that drive regenerative mechanisms (Xie et al. 2007). Thus, striking a balance between mitigating excitotoxicity and allowing for the temporally distinct, early‐phase excitability, could hold the key to unlocking more effective neural regeneration strategies.

### 4.3 DRG Neuronal Plasticity Involves Alterations in Excitability

Recent work has explored a related line of inquiry and demonstrated that in vitro, DRG neurons undergo transcriptional de‐maturation by downregulating genes critical for synaptic transmission (Hilton et al. 2022). Genetic deletion of core components of the synapse essential for neurotransmission—such as RIM1/2 or Munc13—significantly enhances axon growth and regeneration while reducing branching (Hilton et al. 2022). Together with the findings presented here, these results suggest that inflammation‐induced molecular cascades that promote neurite outgrowth are inexorably linked with changes in neural excitability and activity.

Activity‐dependent phenomena are well‐documented in regenerative contexts. Although it is rare for a neuron to lose all input in vivo, as it does in a dissociative culture system, evidence indicates that peripheral neurons may atrophy or even die following long‐term sensory deprivation (Lyu et al. 2017; Zhang et al. 2018; Shifman, Zhang, and Selzer 2008). In this context, it is possible that pain after injury plays a crucial role in promoting the proper re‐maturation of healthy neuronal circuitry. Therefore, the excessive use of analgesics in clinical settings may potentially hinder regenerative outgrowth (Berthézène et al. 2021; Huss, Felt, and Pacharinsak 2019). The experience of pain may be a necessary part of the healing process; acknowledging and pursuing this hypothesis may lead to better outcomes.

While the precise mechanism linking excitability and structural plasticity remains unclear, several plausible hypotheses can be tested. One such hypothesis is that ionic gradients along neuronal membranes allow for calcium influx during action potentials. Calcium is a well‐known second messenger and cofactor for many different molecular cascades that promote both the growth of axons but also neuronal sensitization and pain hypersensitivity (Ziv and Spira 1998; Bradke, Fawcett, and Spira 2012). As a driver of calcium currents, inflammation, a central player in injury responses, is well‐characterised in driving calcium currents (Womack, Macdermott, and Jessell 1988; Simonetti et al. 2013; Linhart, Obreja, and Kress 2003). The early increases in excitability may support increased calcium flux and drive the increased growth of female DRG neurons in response to MCM stimulated with TNFα (T‐MCM). Indeed, we find that inhibiting the calcium‐dependent enzyme CamKII can prevent the enhanced growth state promoted by T‐MCM, similar to the effects of blocking neural activity with RTG. Our findings suggest that early calcium flux may serve as a driving mechanism linking inflammatory activity to increased cellular outgrowth. However, the inability to directly link excitability and outgrowth variables over time limits our ability to confirm causation. Thus, we cannot discount the alternative hypothesis that the observed phenomenon represents a correlation between two distinct processes rather than a causal relationship. Activity‐dependent and activity‐independent mechanisms that enhance outgrowth following T‐MCM treatment may overlap with those leading to decreased excitability. While the presented data is not derived from direct repeated measures of single cells, we are unable to refute this alternative interpretation. However, we believe our findings provide a compelling basis for further studies employing calcium imaging at the established time points could characterise the extent of this overlap, clarify the underlying mechanisms and validate our proposed framework.

### 4.4 DRG Neuronal Plasticity: The Relationship Between Excitability and Pain

Plasticity, excitability and pain are interconnected processes in the nervous system. The maturation and maintenance of neuronal circuits involve signalling cascades that regulate the functional component and structural generation and pruning of synapses, membrane potentials and sensory receptor insertion. These processes involve different cell types, including glia (Wilton, Dissing‐Olesen, and Stevens 2019) and immune cells (Macht 2016), and are activity driven (Yap and Greenberg 2018; Martini et al. 2021). The consequences of these plastic processes can be adaptive or maladaptive, such as sickness behaviour and chronic pain. Pain is one of the most common outcomes after injury and inflammation and has been widely reported in the animal model of neuroinflammatory disease, EAE (Murphy et al. 2020; Segal et al. 2020; Olechowski, Truong, and Kerr 2009; Thorburn et al. 2016). Interestingly, pain in the EAE model is also associated with significant changes occurring at the level of DRG, including increased inflammation and increased neuronal activity/hyperexcitability (Yousuf et al. 2020, 2019). Our lab has also recently reported that common markers associated with increased neural plasticity and growth are increased in DRG neurons of EAE mice in a sex‐specific manner, with females exhibiting higher levels of ATF3 and pCREB (Maguire et al. 2022). Taken together with the results of the experiments described here, we can speculate that DRG neurons from female mice may use the inflammatory stimulation as a priming signal to trigger a change in their growth status. Our study on EAE primarily focuses on the peripheral nervous system, encompassing not only the cell bodies of peripheral sensory neurons but also their axons and terminals. While our in vitro findings from dissociated dorsal root ganglia (DRG) cell bodies align with our EAE results, further investigation into other components of the peripheral nervous system is warranted. Such studies would help determine whether the observed changes in activity and plasticity are confined to specific neuronal compartments. While our results with RTG in the EAE model suggest that inhibiting this increase in neural activity in response to inflammation may be beneficial for pain in the short term, how this treatment affects the long‐term plasticity of these neurons remains to be determined. Additional studies investigating its effects on mechanical hypersensitivity in naïve control mice are also indicated to determine whether the action of RTG is dependent on inflammatory immune activity or functions through independent pathways in sensory neurons.

## 5 Conclusions

The relationship between neuronal excitability and structural plasticity is complex and dynamic, with evidence suggesting that they may be inextricably linked. Both in vitro and in vivo studies have demonstrated the importance of activity‐dependent phenomena in promoting regenerative processes and maintaining nervous system function. The role of pain in this process is an intriguing area of investigation, with some evidence suggesting that it may be necessary for proper re‐maturation of healthy circuitry. The mechanisms underlying the link between excitability and plasticity remain unclear, but the role of Ca^2+^ as a second messenger and cofactor for molecular cascades presents a plausible hypothesis that warrants further investigation. While our findings highlight an effect in the female paradigm, it is critical to examine potential sex differences, and it remains an open question whether this phenomenon operates similarly in the male system. Overall, a deeper understanding of the relationship between neuronal excitability and structural plasticity has important implications for the development of new strategies to promote nerve regeneration and functional recovery after injury or disease.

## Author Contributions

**Timothy N. Friedman:** conceptualization, investigation, formal analysis, writing – original draft. **Shawn M. Lamothe:** investigation, writing – review and editing. **Aislinn D. Maguire:** investigation. **Thomas Hammond:** investigation. **Gustavo Tenorio:** investigation. **Brett J. Hilton:** conceptualization, writing – review and editing. **Jason R. Plemel:** conceptualization, writing – review and editing. **Harley T. Kurata:** conceptualization, writing – review and editing, supervision. **Bradley J. Kerr:** conceptualization, funding acquisition, writing – review and editing, project administration, supervision.

## Ethics Statement

All animal experiments were performed according to Canadian Council on Animal Care's Guidelines and Policies with approval from University of Alberta Health Sciences Animal Care and Use Committee.

## Conflicts of Interest

The authors declare no conflicts of interest.

### Peer Review

The peer review history for this article is available at https://www.webofscience.com/api/gateway/wos/peer‐review/10.1111/jnc.16292.

## Supporting information


**Figure S1.** Conditioned media (MCM, T‐MCM), retigabine (RTG), and KN93 do not increase cell death.
**Table S1.** Summary of antibodies and reagents used in immuno‐histo/cyto‐chemistry experiments.

## Data Availability

A preprint of this article was posted on ResearchSquare; 15th of March 2024; https://www.researchsquare.com/article/rs‐4094312/v1. The datasets supporting the conclusions of this manuscript are available upon request.
