# Supplementary material for: Plasticity of Mouse Dorsal Root Ganglion Neurons by Innate Immune Activation Is Influenced by Electrophysiological Activity
Source: J Neurochem. 2024 Dec 26;169(1):e16292. doi: 10.1111/jnc.16292 (PMC11671441; doi:10.1111/jnc.16292)
Supplement: Supplementary file 1 — Figure S1. Conditioned media (MCM, T‐MCM), retigabine (RTG), and KN93 do not increase cell death. Table S1. Summary of antibodies and reagents used in immuno‐histo/cyto‐chemistry experiments. [file JNC-169-0-s001.pdf]

Plasticity of mouse dorsal root ganglion neurons by innate immune activation is influenced by electrophysiological activity

Timothy N. Friedman[1#], Shawn M. Lamothe[2], Aislinn D. Maguire[1], Thomas Hammond[2], Gustavo Tenorio[5], Brett J. Hilton[3], Jason R. Plemel[1,4], Harley T. Kurata[2], and Bradley J Kerr[1,2,5*]

Supplemental Material

Supplementary Table 1.

	Target	Species	Dilution	Supplier	Product Code	RRID
Primary Antibodies	BIII Tubulin	rb anti-hu	1:1000	Sigma	T2200	AB_262133
Secondary Antibodies	488-IB4	N/A	1:100	Fisher	I21411	
	594	dk anti-rb	1:500	Jackson ImmunoResearch	711-586-152	AB_2340622
	DAPI	N/A	1:1000	Invitrogen	D1306	
Cell Culture Reagents	24 well, Tissue Culture Treated Plate			CellVis	P24-1.5H-N	
	24 well, Tissue Culture Treated Plate			Falcon	353047	
	15mm Glass Coverslips			Fisher	1254583	
	Poly-D-lysine			Sigma	P6407	
	DMEM -/- high glucose			Gibco	11960069	
	Sodium Pyruvate			ThermoFisher	11360070	
	Glutamax			ThermoFisher	35050061	
	Pen/Strep			Gibco	15140-122	
	FBS			Gibco	12483020	
	Stemxyme I			Worthington	LS004106	
	DNase			Worthington	LS002007	
	Low Ovomucoid			Worthington	LS003086	
	Normal Donkey Serum			Sigma	566460	
	70 um mesh filter			Biologix	15-1070	
	BSA for 20% solution			Sigma Aldrich	A7906	
	BSA for 0.5% solution			Sigma Aldrich	A4161	
EAE Reagents	MOG_{35-55} in CFA (Hook Kits^{TM})			Hooke Laboratories	EK-2110	
	Pertussis Toxin, *Bordatella pertussis*			Hooke Laboratories	BT-0105	
Pharmacological Reagents						
	TNFα			R&D Biosciences	410-MT	
	Retigabine (RTG)			Tocris	6233	
	KN93			Tocris	1278	
Equipment	Axopatch 200B amplifier			Molecular Devices		
	Digidata 1440 digitizer			Molecular Devices		
	Clampex10			Molecular Devices		
	Clampfit 10.7			Molecular Devices		
	ImageJ			NIH		

Supplementary Figure 1

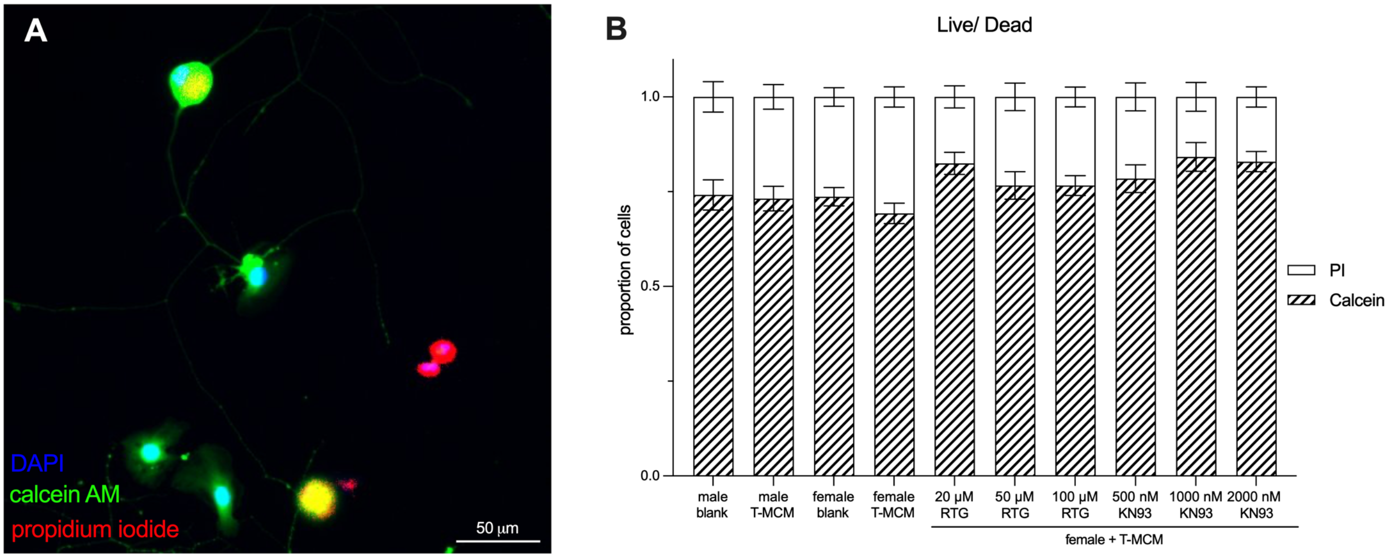

Conditioned media (MCM, T-MCM), retigabine (RTG), and KN93 do not increase cell death. (A) Representative image of DRG culture stained with DAPI (blue), calcein AM (green) and propidium iodide (red). Graph represents proportion of total DAPI-positive cells stained with either calcein AM (live cells) or propidium iodide (dead cells) ± SEM, n=8 across two experiments.
